# Effect of early versus delayed cord clamping in neonate on heart rate, breathing and oxygen saturation during first 10 minutes of birth - randomized clinical trial

**DOI:** 10.1186/s40748-019-0103-y

**Published:** 2019-05-30

**Authors:** Ashish KC, Nalini Singhal, Jageshwor Gautam, Nisha Rana, Ola Andersson

**Affiliations:** 1 Department of Women’s and Children’s Health, Uppsala University, Uppsala, Sweden; 2 University of Calgary, Calgary, Canada; 3 Paropakar Maternity and Women’s hospital, Kathmandu, Nepal; 4 Department of Clinical Sciences Lund, Pediatrics/Neonatology, Skane University Hospital, Lund University, Lund, Sweden

**Keywords:** Delayed cord clamping, Term and late preterm infants, Heart rate in first 10 min, Oxygen saturation in first 10 min, Spontaneous breathing, Randomized clinical trial

## Abstract

**Background:**

Delayed cord clamping (DCC) after 180 s reduces iron deficiency up to 8 months of infancy compared to babies who received Early Cord Clamping (ECC) at less than 60 s. Experimentally DCC has shown to improve cardio-vascular stability. To evaluate the effect of delayed (≥180 s) group versus early (≤60 s) cord clamping group on peripheral blood oxygenation and heart rate up to 10 min after birth on term and late preterm infants.

**Methods:**

We conducted a single centred randomized clinical trial in a low risk delivery unit in tertiary Hospital, Nepal. One thousand five hundred ten women, low risk vaginal delivery with foetal heart rate (FHR) ≥ 100 ≤ 160 beats per minute (bpm) and gestational age (≥33 weeks) were enrolled in the study. Participants were randomly assigned to cord clamped ≤60 s of birth and ≥ 180 s. The main outcome measures were oxygen saturation, heart rate from birth to 10 min and time of spontaneous breathing. The oxygen saturation and heart rate, the time of first breath and establishment of regular breathing was analysed using Student t-test to compare groups. We analysed the range of heart rate distributed by different centiles from the time of birth at 30 s intervals until 10 min.

**Results:**

The oxygen saturation was 18% higher at 1 min, 13% higher at 5 min and 10% higher at 10 min in babies who had cord clamping in delayed group compared to early group (*p* < 0.001). The heart rate was 9 beats lower at 1 min and3 beats lower at 5 min in delayed group compared to early group (p < 0.001). Time of first breath and regular breathing was established earlier in babies who had cord clamping at 180 s or more.

**Conclusion:**

Spontaneously breathing babies subjected to DCC have higher oxygen saturation up to 10 min after birth compared to those who have undergone ECC. Spontaneously breathing babies with DCC have lower heart rates compared to ECC until 390 s. Spontaneously breathing babies receiving DCC have early establishment of breathing compared to ECC.

**Trial registration:**

ISRCTN, 5 April 2016.

## Background

At the time of birth, the pulmonary and cardio-vascular transition from intra-uterine to extra-uterine life depends on two major physiological events, commencement of breathing and transition from dependence of the blood flow through the umbilical circulation [1]. Disturbance in anyone of the functions can result in hypoxia which can progress to an ischemic insult and death [2, 3].

The trigger of breathing at birth results in absorption of the liquid in the trachea and pulmonary airways reducing the pulmonary vascular resistance, aeration of lungs and increase in pulmonary blood flow [4, 5]. This increase in pulmonary blood flow increases the left ventricular preload.

In the feto-placental state, cardiac output from the right ventricle in the foetus bypasses the lungs and flows from the main pulmonary artery and into the descending aorta via the ductus arteriosus [1]. As a result, blood flows continuously through the ductus arteriosus, by-passing the pulmonary circulation into the systemic circulation as right to left shunting.

When umbilical cord clamping (CC) takes place, the umbilical venous blood supply is disrupted with the interruption of the left ventricular pre-load and thus cardiac output [1]. Cardiac output remains low until the lung aeration and pulmonary blood flow (PBF) increases the venous return and preload for the left ventricle and possibly the right ventricle via the foramen ovale [1]. When PBF increases before umbilical CC, the supply of ventricular preload can immediately switch from umbilical to pulmonary venous return without diminution of supply [1]. Due to evidence shown, recent guidelines for active management of the third stage of labour no longer recommend early CC, but changes in practice are still questioned and policies in hospitals are rare [6–8].

Delayed CC allows time for a transfer of the foetal blood in the placenta to the infant at the time of birth [9]. This placental transfusion can provide the infant with an additional 40% more blood volume [10]. The amount of blood transferred to the infant depends on when the cord is clamped and with respect to the height above or below the placenta or the heart of the mother the infant is held prior to clamping [11]. Neonatal benefits associated with this increased placental transfusion include higher haemoglobin concentrations, additional iron stores and less anaemia in infancy and better cardiopulmonary adaptation [4, 12–14]. Delayed CC is also associated with improved developmental milestones at infancy until 4 years of age [15].

An observational study on 109 home births where delayed CC was performed indicated a higher SpO_2_ combined with a lower HR during the first minutes [16]. In the first minutes after birth, tachycardia (HR > 180 bpm) occurred less often, and bradycardia (< 80 bpm) more often compared with previously defined reference ranges obtained from newborns after early CC [17, 18].

We hypothesized that by facilitating umbilical venous return after birth as a result of delayed cord clamping, ventricular preload and cardiac output would improve. We conducted a randomized controlled trial to assess the effects of delayed (≥180 s) versus early (≤60 s) cord clamping on peripheral blood oxygenation and heart rate up to 10 min after birth on term infants.

## Methods

Setting-The study was conducted at Paropakar Maternity and Women’s Hospital (PMWH), a tertiary government hospital providing gynaecological and obstetrics services in Kathmandu, Nepal between April and September 2016. The hospital provided delivery services to 18,567 women in 2016. In 2016, there were 9/1000 early neonatal mortality cases and 19/1000 stillbirth cases, giving a perinatal mortality rate of 28/1000 [19].

Study design-A randomized, controlled design was used. Participants were randomly assigned to one of two parallel groups with a 1:1 ratio, with one group receiving cord clamping at less than 60 s after birth (ECC) and the second group receiving cord clamping at 180 s or more (DCC).

### Participants

#### Eligibility criteria for the women

Pregnant women not in labour with foetal heart sound at admission, those with no medical or obstetrical complication during pregnancy and women who consent to participate in the study.

#### Inclusion criteria

Women admitted in the low risk delivery unit for delivery, normal vaginal delivery, women with no complication during delivery, foetal heart rate (FHR) ≥100 ≤ 160 bpm and women with gestational age (≥33 weeks).

#### Exclusion criteria

Women who have stillbirth, congenital anomaly, Rh-incompatibility, multiple gestation and non-breathing babies.

**Fig. 1:**
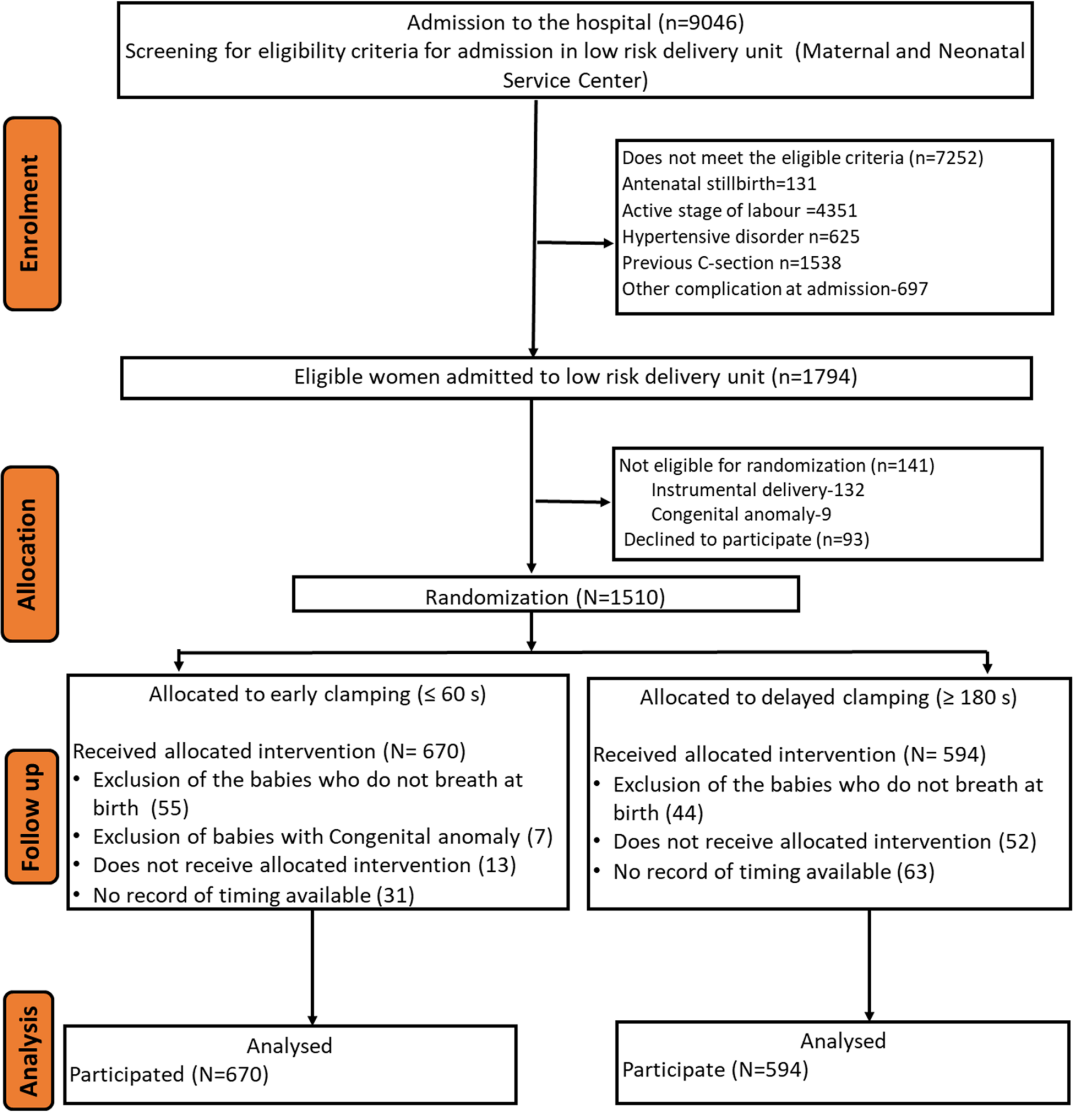
Trial profile (CONSORT Flow chart)

If the baby was not breathing within 30 s, the standard protocol for resuscitation as per the Helping Babies Breathe guideline was provided, where in the cord was immediately cut and the baby taken to resuscitation table for resuscitation (Fig. 1). Babies who received stimulation, suctioning or bag-and-mask ventilation were excluded. The heart rate was not used for determining the resuscitation to the baby.

Randomization-The principal investigator (AKC) prepared a random list using random digit generator and had no further clinical involvement in the trial. Following this, AKC prepared sequentially numbered, opaque envelopes and put in colour cards with details of the allocated group and sealed the envelopes. These were kept at the research unit and were brought in the delivery unit before randomization.

### Intervention

Randomization and treatment group allocation took place a few minutes before delivery when the nurse midwife considered that vaginal delivery was imminent. To allocate the women into treatment group, research surveillance officers (SO) opened the next consecutively numbered envelope and informed the nurse-midwife of the assigned treatment. The used colour card and envelope were discarded.

The SO used a stop watch to measure the time from complete delivery of the baby until the first umbilical cord clamp. All other aspects of obstetric care were managed according to standard practice at the hospital. In both groups, oxytocin was provided after the umbilical cord was clamped. All staff in the delivery unit were trained in the study procedures before the trial started.

The research surveillance team consisted of trained nurse-midwives with at least 1 year of experience in clinical research. A 10-day training was provided to the research team on selection criteria of women, taking consent, placing Moyo® foetal heart rate monitor-FHRM (Laerdal, Norway) and placement of neonatal probe of a Masimo® pulse oximeter (Masimo Corporation, Switzerland) in the infant’s right thumb immediately after birth until 10 min. The SO measured the time of initiation of breathing and establishment of regular breathing.

### Outcome measure

- Oxygen saturation at 1, 5 and 10 min
- Heart rate measured at 1, 5 and 10 min
- Heart rate measured continuously from time of birth to 10 min
- Initiation of spontaneous breathing and establishment of regular breathing.

### Data collection

A research surveillance team was established at the admission, antenatal, and delivery units for the enrolment of the participants and collection of data. The (SO collected background information on the women from the hospital records. The FHRM was placed in the women’s abdomen during the transfer and where it remained there until delivery. A SO was dedicated to each woman to monitor the FHR and exclusion would be made if the FHR became abnormal. Immediately after delivery, another SO placed FHRM over the left chest of the baby to measure the heart rate from birth until 10 min, she also, as soon as possible, placed the pulse oximeter in the right thumb until 10 min. The average time for placing FHR in baby’s chest and pulse oximeter in the right thumb was 30 and 45 s respectively. For both the treatment group, the Apgar score was taken at 1 and 5 min using the stop watch. The temperature of the baby was measured during the first 30 min. The health status of the baby at discharge was documented.

### Blinding

The women participating in the study were blinded on the treatment allocation to the extent possible. Ethical approval was received from the Nepal Health Research Council (ref: 92/2015) and was registered as clinical trial (10.1186/ISRCTN10944304). Informed written consent was taken from the women who enrolled in the study.

### Sample size

The systematic review by Fleming et al. on the HR at birth has concluded that at birth the median heart rate is 127 bpm with 1st centile HR 90 and 99th centile HR 164. The 3 SD is estimated to be 37 bpm with 1 SD 12.3 bpm [20]. Using this as reference, for this study, we calculated as following:

A power analysis shows that a group size of 566 would allow us to find a difference of 5 in heart rate between groups at 2 min after birth with a power of 80% and a significance level of 0.05, assuming a mean (SD) heart rate of 140 (30) in the DCC group. Allowing for an attrition of 25%, we planned to include 755 participants in each group. http://www.sample-size.net/sample-size-means/ calculator was used to calculate the sample size.

### Data analysis

We conducted the comparison of the background characteristics between the treatment groups. Student’s t-test was used for continuous variables with normal distribution such as maternal age, gestational age and birth weight. For background characteristics such as complication during pregnancy with skewed distribution we used Mann Whitney U test. For the number of previous pregnancies with median, we used Fisher’s exact test. The oxygen saturation and heart rate, the time of first breath and establishment of regular breathing, and Apgar score was analysed using Student t-test to compare groups. The correlation between the pulse oximeter and FHRM was compared using Pearson’s correlation test. We analysed the range of heart rate distributed by different centiles from the time of birth at 30 s intervals until 10 min. Only cases handled according per protocol were included in the analysis.

## Results

During the study period, 9046 women were admitted to the hospital for delivery. Of these, 1794 met the eligibility criteria of low risk delivery. A total of 141 were not included for randomization as these women were referred for instrumental delivery or had congenital anomaly; 93 declined to participate. A total of 1510 were included for randomization. Of the 755 allocated for ECC, 89.3% received the allocated intervention. Of the total 755 allocated for DCC, 78.9% received the allocated intervention. For the analysis, 670 ECC and 594 DCC babies who adhered to the protocol were taken for consideration (Fig. 1).

We found differences in the maternal age and complications during pregnancy between the treatment groups. (Table 1). Significant factors that differed between the two groups were included in regression analysis for adjustment. After adjustment, we found mothers age and complication at birth had no significant effect on heart rate. Even after adjustment, the treatment allocation had effect on heart rate (Table 2).

**Table 1:** Background characteristics of two treatment groups

Characteristics	Cord clamping at < 60 s (*n* = 670)	Cord clamping at ≥180 s (*n* = 594)	*p*-value
Time to clamp cord (mean ± SD) (in seconds)	31.2 ± 14.4	198.5 ± 16.9	–
Mother’s age (in years)	24.0 ± 4.3	23.1 ± 3.9	< 0.001^a^
Gestational age (in weeks)	39.4 ± 1.3	39.4 ± 1.3	0.69^a^
Number of pregnancies including present in Median (Min, Max)	1 (1, 8)	1 (1, 4)	0.006^b^
Complication detected during pregnancy	19.0 (2.8%)	15.0 (2.5%)	0.86^c^
Birth weight (in grams)	3011 ± 382	3079 ± 393	0.002^a^

Result is presented in mean ± standard deviation, count (proportions), or median (minimum, maximum)

^a^t-test applied

^b^Mann-Whitney U test applied

^c^Fisher’s exact test applied

**Table 2:** Linear regression between background characteristics and treatment outcome with POX Heart rate reading at 1 min as dependent variable

	Std. Error	Beta coefficient	*p*-value
(Constant-HR at 1 min (pulse oximeter)	2.225	116.228	< 0.001
Mothers age	0.034	−0.008	0.713
Randomization	0.283	−0.689	< 0.001
Complication detected during pregnancy	0.879	0.004	0.832
Birth weight (grams)	0.000	−0.001	0.964

The oxygen saturation was 18% higher at 1 min, 13% higher at 5 min and 10% higher at 10 min in babies who had cord clamping in the delayed group compared to the early group (*p* < 0.001). The heart rate was 9 beats lower at 1 min and 3 beats lower at 5 min in delayed group compared to early group (p < 0.001). Time of first breath and regular breathing were established earlier in babies who had cord clamping at 180 s or more. The Apgar score was better at 1, 5 and 10 min in the DCC than ECC group (Table 3). There were strong correlations between heart rate measured by pulse oximeter and FHRM at 1, 5 and 10 min (Appendix).

**Table 3:** Oxygen saturation, heart rate and at 1, 5 and 10 min and Breathing in treatment groups

	Cord clamping at < 60 s (*N* = 670)	Cord clamping at ≥180 s (*N* = 594)	Mean difference
Oxygen saturation at 1 min	61.3 ± 5.5	79.8 ± 3.5	−18.4 (− 17.9,-18.9)
Oxygen saturation at 5 min	77.9 ± 6.5	91.2 ± 3.1	−13.2 (− 12.6,-13.8)
Oxygen saturation at 10 min	87.7 ± 3.4	98.0 ± 1.4	−10.3 (− 10.3,-10.6)
Heart rate at 1 min	116.2 ± 4.1	106.8 ± 5.8	9.4 (10.0, 8.9)
Heart rate reading at 5 min	134.3 ± 2.9	131.6 ± 4.9	2.8 (3.2, 2.4)
Heart rate reading at 10 min	136.3 ± 1.9	137.2 ± 2.1	−0.9 (− 0.7,1.1)
Time of first breath (in seconds)	14.5 ± 7.6	10.4 ± 7.2	4.2 (5.0, 3.3)
Time of regular breathing (in seconds)	19.0 ± 13.1	14.9 ± 13.0	4.1 (5.5, 2.7)
Apgar score at 1 min	7.4 ± 0.9	8.1 ± 0.6	−0.6 (−0.5, − 0.7)
Apgar score at 5 min	9.0 ± 0.6	9.1 ± 0.7	−0.1 (− 0.1, − 0.2)
Apgar score at 10 min	9.9 ± 0.3	10.0 ± 0.2	−0.04 (− 0.01, − 0.1)

**Fig. 2:**
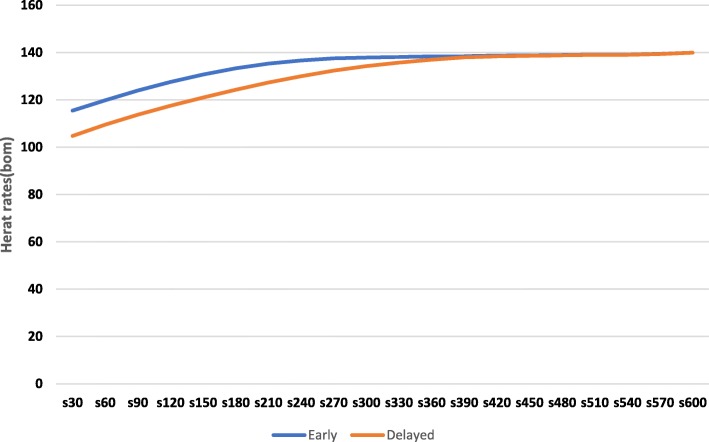
Heart rate trends over time in early and late cord clamped groups

The continuous heart rate measurement demonstrated lower heart rate in DCC until 390 s and after that the heart rate in the two treatment group plateaued. (Fig. 2).

With the intention to treat, the findings were similar (Appendix).

## Discussion

This study on babies with spontaneous breathing at birth reports higher oxygen saturation at 1, 5 and 10 min in babies who had DCC than those who had ECC and earlier establishment of breathing and regular breathing in babies who had DCC at birth. There is a lower heart rate in babies who had DCC than those who had ECC up until 6 min after delivery.

Bhatt et al. Two thousand thirteen reported that clamping preterm lambs before ventilation had reduced heart rate^4^ in contrast to Polglase et al’s experimental studies in preterm lambs, which showed rising heart rate and rapid decrease in oxygen saturation in the early clamped group [21].

The observational study in newborns by Smith et al. Two thousand fourteen also showed early clamping resulted in a higher heart rate [16]. Findings of Polglase et al. and Smith et al. was concurrent with our findings on higher oxygen saturation and lower heart rate in the first 5 min of birth in the DCC group [16, 21]. Katheria et al. conducted a feasibility trial on measuring cardiac changes during delayed cord clamping after vaginal delivery in term infant. Cardiac output increased from 2 to 5 min of birth [22].

The possible mechanism for HR < 100 bpm at the first minute of life due to reflex bradycardia might have misled clinicians to diagnose the case as requiring some assistance at birth and might have overestimated the number of babies requiring resuscitation [23].

The updated 2015 guideline by International Liaison Committee of Resuscitation (ILCOR), warrants need to start positive pressure ventilation if the heart rate is less than 100 beats per min (bpm) at birth [24]. Heart rate (HR) is the most important, objective clinical indicator of the health of newly born infants [25, 26]. Increasing HR is considered to be a good marker of effective resuscitation, and a HR exceeding 100 bpm is considered normal [27]. The evidence for setting 100 bpm as cut off for initiating neonatal resuscitation is arbitrary and has poor quality evidence [23]. Determining HR immediately after birth is usually done by auscultation or palpation of the umbilical cord [28]. A study by Dawson et al. has shown that the median HR was < 100 bpm at 1 min [18]. The low HR in the first minutes after birth may be physiological in some infants yet the standard teaching is that infants with a HR < 100 bpm after birth should receive positive-pressure ventilation [18].

There are two mechanisms responsible for the bradycardia, one reflex due to vagal stimulation and the other a direct effect of asphyxia in the cardiac muscle [29]. Bradycardia after birth is considered to be due to vagal stimuli, especially if the cord is cut before the infant has taken a breath [29, 30]. Intrauterine hypoxia and acidosis may be the cause of bradycardia at birth in some infants [1, 29].

Some of the limitations of the study are correlated to the difficulties associated with studies conducted in high volume delivery settings. Inclusion was performed during a period of 6 months, which might prevent biases that could occur due to a longer inclusion period. Furthermore, there was a high protocol deviation in the delayed cord clamped group. As analysis was made by intention to treat, 25% of the infants reported in the delayed cord clamped group were clamped before 1 min, as the nurse-midwives perceived that early clamping against the allocation was required.

## Conclusions

In this study we show that DCC for 180 s was an effective intervention to ensure higher oxygen saturation at 1, 5 and 10 min, lower heart rate at 1 and 5 min and early establishment of spontaneous breathing. There is a lower heart rate in babies who had DCC than those who had ECC up until 6 min after delivery.

## Appendix

**Table 4:** Correlation between Moyo's heart rate and POX heart rate at different time interval

Time	N	Pearson Correlation	Sig. (2-tailed)
At 1 min	1270	0.987^a^	<0.001
At 5 min	1264	0.964^a^	<0.001
At 10 min	1264	0.834^a^	<0.001

^a^Correlation is significant at the <0.01 level (2-tailed)

## Abbreviations

BPM: Beats per minute
DCC: Delayed Cord Clamping
ECC: Early Cord Clamping
FHR: Fetal Heart Rate
HR: Heart Rate
ILCOR: International Liaison Committee of Resuscitation
SD: Standard Deviation
SO: Surveillance Officer
